# Adding body load modifies the vibratory sensation of the foot sole and affects the postural control

**DOI:** 10.1186/s40779-018-0175-4

**Published:** 2018-08-17

**Authors:** Yves Jammes, Eva Ferrand, Corentin Fraud, Alain Boussuges, Jean Paul Weber

**Affiliations:** 1 School of Podiatry, 13014 Marseille, France; 2 C2VN Inra Inserm, Faculty of Medicine, Aix Marseille University, Bd. Pierre Dramard, 13916 cedex 20, Marseille, France

**Keywords:** Foot sole sensitivity, Vibration, Weight carrying, Postural control

## Abstract

**Background:**

Heavy backpacks are often used by soldiers and firefighters. Weight carrying could reduce the speed and efficiency in task completion by altering the foot sole sensitivity and postural control.

**Methods:**

In fifteen healthy subjects, we measured the changes in sensitivity to vibrations applied to the foot sole when standing upright or walking after load carrying (30% body weight). The participants were asked to judge different vibration amplitudes applied on the 2nd or 5th metatarsal head and the heel at two frequencies (25 and 150 Hz) to determine the vibration threshold and the global perceptual representation (Ѱ)of the vibration amplitude (Ф) given by the Stevens power function (Ѱ = k × Ф^n^). Any increase in negative k value indicated a reduction in sensitivity to the lowest loads. Pedobarographic measurements, with computation of the center of pressure (COP) and its deviations, were performed during weight carrying.

**Results:**

The 25-Hz vibration threshold significantly increased after weight carrying when standing upright or walking. After standing with the added loads, the absolute negative k value increased for the 25 Hz frequency. After walking with the added loads, the k coefficient increased for the two vibration frequencies. Weight carrying significantly increased both the CoP surface and CoP lateral deviation.

**Conclusions:**

Our data show that weight carrying reduces the sensory pathways from the foot sole and accentuates the center of pressure deviations.

## Background

Heavy backpacks are often used by soldiers and firefighters. Previous studies have focused on the consequences of weight carrying on postural control*.* Majumdar et al. [1] showed that load carrying by soldiers (reaching up to 27% of the body weight) affected kinematics of the gait. Another study [2] reported that load carrying by soldiers also increased the energy cost of walking. Studies have shown that carrying a military backpack of 18 kg [3] or a firefighter equipment [4, 5] can reduce standing balance. Park et al. [4] reported a decrease in anterior-posterior and medial-lateral excursion of the center of plantar pressure (COP) trajectory during walking with decreased COP velocity and increased foot-ground contact time and stride time. A decreased upper extremity sensation was also reported during heavy loads carrying [6], and this sensation was associated with a reduced blood flow, which is suspected to produce neurological dysfunction. No data were found on an altered foot sole sensation after overload carrying and the accompanying changes in postural control. Indeed, the compression of epidermal and dermal foot layers during weight carrying, as well as the resulting changes in mechanosensitivity, could persist after the removal of overload equipment.

The cutaneous mechanoreceptors of the foot sole detect changes in the application of the mechanical loads on the plantar surface during gait and standing and contribute to controlling the standing balance and postural reflexes in healthy subjects [7–9]. The foot sole cutaneous afferents respond differently across vibration frequencies [10–12]. The fast adapting type II Meissner endings are sensitive to dynamic skin deformation of relatively low frequencies (5–50 Hz) whereas the Pacinian endings are sensitive to high frequencies. The sensitivity of the foot sole afferents could depend on their location in the skin and their proximity to the epidermal layer [13, 14]. They are influenced by the mechanical properties of the skin which vary with calluses [15] and probably also with weight carrying as suggested by Mildren et al. [16], who found that the foot sole skin vibration perceptual thresholds are elevated during standing compare to sitting. Another study by Lhomond et al. [17] reported that wearing a loaded vest reduced the somatosensory cortical potentials evoked by electrical stimulation applied under the sole of the foot. The type of feedback thought to be transmitted by the different types of foot sole receptors differs between the slow and fast afferent units. Slowly adapting afferents are thought to provide pressure feedback which will be certainly affected by weight bearing, but fast adapting afferents may play a larger role in detecting and modulating responses to slips across the skin as those present during gait [11]. The activation of the cutaneous afferents gives perceptual information that reveals relationships between the estimate of the vibratory stimuli and their physical magnitude. This was confirmed for the mechanosensitivity of the hand [18] and the foot sole [15, 19, 20].

Because the perception of vibration applied on the foot sole cannot be explored during weight carrying sessions, the core of the present study was related to the after effect of carrying overload equipment when standing upright or walking. We hypothesized that load-induced changes in the foot sole sensitivity could persist following removal of the load. We considered the changes in vibration threshold and global perception of added loads provided by the Stevens power function. Previous studies only reported changes in the vibration threshold [16], but not in global perception. Pedobarographic measurements were also performed on separate days during the same week to confirm that weight carrying modified postural control. No data are reported on the variations of postural control after weight carrying.

## Methods

### Subjects

Fifteen healthy young subjects (7 females) (mean age: 24 ± 2 years; mean weight: 70 ± 5 kg) were studied. All were free of foot pain and had no history of trauma or surgery of the feet or legs. None were involved in an exercise program. All subjects had normal detection thresholds of the foot sole to light touch measured with Von Frey monofilaments. All patients provided informed consent, and the study was approved by the Ethics Committee for Human on September 9th, 2015. N° 2014-AO1969–38.

### Measurements of vibratory sensation

The skin sensitivity was evaluated using vibration testing at two frequencies (25 and 150 Hz) at each one of two plantar locations (2nd or 5th metatarsal head and the heel). The method has been described in detail in our previous studies [15, 19]. The vibration frequencies of 25 Hz and 150 Hz were chosen in an attempt to respectively activate the Meissner endings (25 Hz) or the Pacinian endings (150 Hz) [11, 12]. Sinusoidal vibrations were applied to the heel and the 5th metatarsal head via a plastic probe (width: 2 mm; length: 5 mm) attached to a minishaker (model 201, Ling Dynamic Systems, Royston, UK). A preload force of 2 N was applied, manipulated by a vertical adjustment of the shaker and confirmed with a force transducer (Scaime model K13–0.02 kN, Annemasse, France). Our vibrator device allowed for the delivery of 7 different amplitudes of vertical probe motion. The vibration motion, expressed in micrometer (μm), was measured using an accelerometer attached to the probe (MAES France, model EOAS S114 D2500, Les Clayes-sous-Bois, France). The vibration magnitude depended on its frequency and varied in a range of 10 to 360 μm at 25 Hz and 10 to 180 μm at 150 Hz. The testing frequencies were randomized at each foot sole location and the testing order of the foot sole location was also randomized across the participants. In each subject, 4 trials were given at each frequency-location.

### Psychometrical evaluation of sensations

The measurement task for each participant was to judge the magnitude of different vibration amplitudes at each frequency (25 and 150 Hz) which were delivered at random. The judgments of the stimuli were recorded on a 0–10 cm visual analogue scale. The participants’ specific standards for 0 and 10 on this scale were established in pilot tests in which the lowest and the highest stimulus were presented twice in order to acquaint the subjects with the full range of loads. After this acclimatization, the experimenter remained silent during further tests and the participants indicated their estimate immediately after each stimulation. First, the vibration detection threshold was determined in each plantar location by considering the lowest detectable load at each vibration frequency. Second, the Stevens power function (Ψ = k × Φ^n^) allowed for the acquisition of regression equations between the estimate of the vibration stimuli (Ψ) and their physical magnitude (Φ) [21]. The exponent n in the power law was determined by a linear regression analysis between Napierian logarithmic (Ln) transformed stimuli and estimation data. Regressions were obtained for each test performed in each individual and the significance against zero of the R coefficient was tested. The n coefficient is the slope of the regression line obtained between logarithmic transforms of stimuli and estimations, and it measures the changes in perception between the extreme values of loads. All k values were negative, and any increase in absolute value of k indicated a reduced sensitivity to the lowest loads. The scattering of pair values collected for each run was estimated by the standard errors of both the k and n coefficients.

### Weight carrying

A fisherman waistcoat with backpack and thorax pockets was used. Cast iron disks for weightlifting of 2, 5, or 10 kg were inserted in the pockets to reach a total added load of 30% ± 2% of the body weight. Among the subjects, a total of 16 to 28 kg disks was carried during standing or walking.

### Pedobarographic measurements

The subjects were bare-footed and had open eyes when standing in double limb stance on the pedobarographic platform for 30s. The computerized 530 × 600 mm strain-gauge platform (WinPOD, Medicapteurs SA, Toulouse, France) consisted of 2304 resistive load cells and its sampling frequency was 100 images/s. We measured the total plantar contact area of both feet, the peak and mean foot pressures, the surface of the COP (the area described by COP excursions during each 30 s measurement period), and maximal COP excursions (the lateral/medial deviations measured by the computer program of the platform after fixing the reference point at the middle of COP surface).

### Protocol

First, the effects of weight carrying on the vibration perception were studied at the end of the 4 min standing period immediately after the weights had been removed, and the measurements were repeated at the 10th min. Then, we waited for a 30 min rest period without weights. Second, the subjects had to walk 400 m with the weights, and the measurements of vibration sensitivity were performed immediately after the subject had stopped walking and removed the weights. This was repeated 10 min later. In both conditions, control values of vibration sensitivity (threshold, n and k coefficients of the perceptual sensation) were collected before standing and walking sessions. On a separate day within the same week as the vibration perception trials, pedobarographic measurements were taken before and after weight bearing upright stance.

### Statistical analyses

All data in Tables and Figures show the mean ± standard error of mean (SEM). An ANOVA for repeated measures was only used to compare the vibration detection thresholds of the vibratory sensations measured before and after the weight carrying sessions. For the perceptual analyses, regressions between the estimate (Ψ) of vibratory stimuli and their physical magnitude (Φ) were obtained for each test performed in each individual and the significance against zero of the R coefficient was tested. The scattering of pair values collected for each run was estimated by the standard errors of both the k and n coefficients. Differences between regression lines obtained at each foot location and each vibration frequency were assessed by Student’s *t* test comparing the mean and SEM of the n and k coefficients. Significant differences between two successive measurements were considered when the *P* value was < 0.05.

## Results

### Foot sole sensitivity after removal of the load

The vibration sensitivity thresholds were measured before (control) and immediately after standing upright or walking with the added weight (test). Table 1 shows that after weight carrying sessions when standing upright, the threshold for the 25-Hz vibration frequency significantly increased in the 5th metatarsal head and the heel. Ten min following removal of overloads, the reduced sensitivity of the 5th metatarsal head disappeared, and the effect persisted in the heel. After walking with added weight, the threshold for the 25 Hz vibration frequency significantly increased in the heel. No changes were noted at both frequencies in the 5th metatarsal head.

**Table 1:** Vibration thresholds (in μm) measured at the 5th metatarsal head and the heel (mean ± SEM)

Categories	Control	Overload removal	10 min after overload removal
Standing			
5th metatarsal head			
25 Hz	24 ± 3	41 ± 4^(1)^	26 ± 3
150 Hz	22 ± 1	21 ± 0	22 ± 1
Heel			
25 Hz	30 ± 6	43 ± 6^(1)^	37 ± 7^(1)^
150 Hz	20 ± 0	22 ± 1	20 ± 0
Walking			
5th metatarsal head			
25 Hz	27 ± 5	30 ± 6	37 ± 7
150 Hz	24 ± 1	24 ± 0	25 ± 3
Heel			
25 Hz	37 ± 7	51 ± 7^(1)^	27 ± 5
150 Hz	20 ± 0	20 ± 0	24 ± 3

**Fig. 1:**
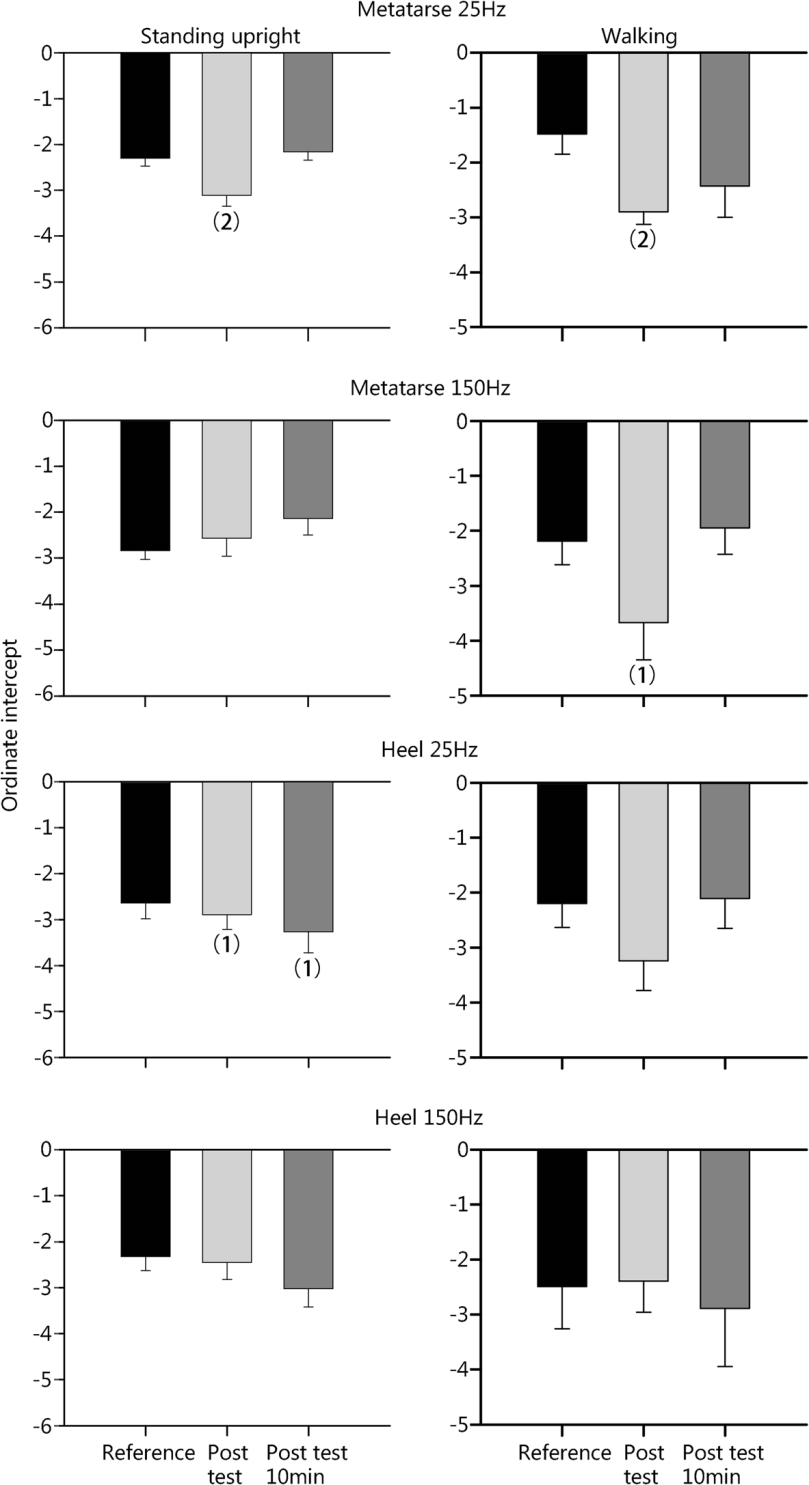
The changes in k coefficient of Stevens power function after weight carrying during standing upright (left) or walking (right). Values are the mean ± SEM (Standard error of mean). (1) *P* < 0.05; (2) *P* < 0.01

**Fig. 2:**
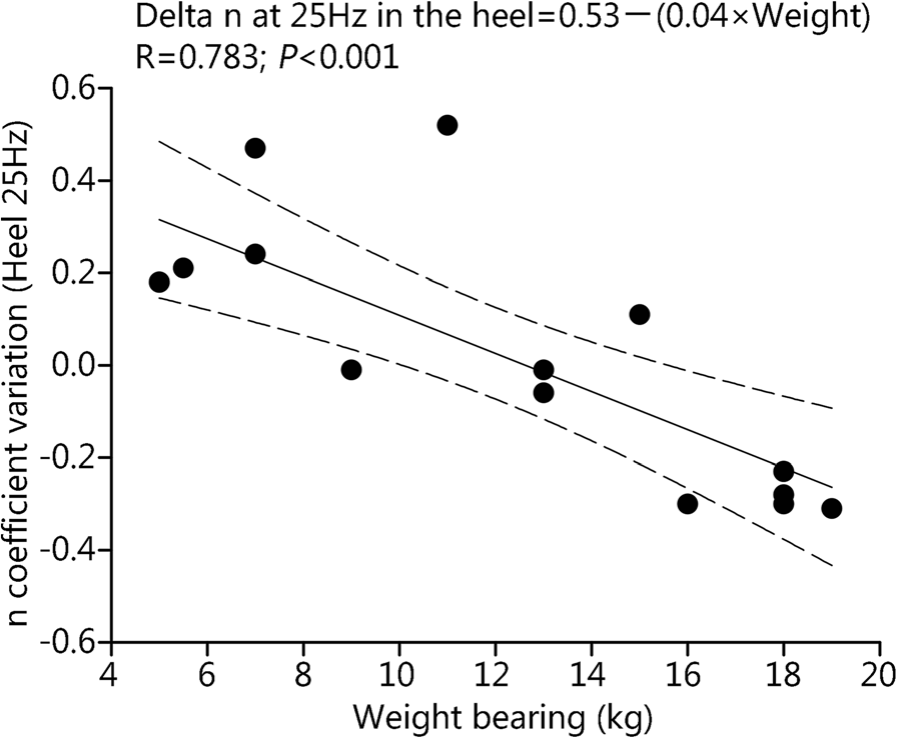
After weight carrying when standing upright, the n coefficient of the Stevens power function was negatively correlated with the individual value of overloads. The changes were only noted for the perception of 25 Hz vibration frequency in the heel location. The regression line with 95% confidence interval is shown

*SEM* Standard error of mean; ^(1)^*P* < 0.05;

After standing with added loads (Fig. 1), the absolute negative value of the k coefficient of the Stevens power function increased in both foot locations at the 25-Hz frequency, and not the 150 Hz frequency, indicating a reduced sensation for the lowest vibration amplitudes. Ten min after removal of the weight, these changes in k coefficient persisted for the heel but not for the 5th metatarsal head. The n coefficient of the Stevens power function obtained at 25 Hz in the heel was negatively correlated with the magnitude of added loads carried by the different subjects (Fig. 2).

After walking with added loads (Fig. 1), the k coefficient only increased in the 2nd metatarsal head at the two vibration frequencies. The changes were no more significant 10 min following removal of the load. No changes in the n coefficient were found (Table 2). Notably, the n values measured at the 150 Hz frequency were always significantly higher than those measured at 25 Hz.

**Table 2:** Exponent (n) of the Stevens power function measured (mean ± SEM)

Categories	Control	Added overload	Added overload 10 min later
Standing			
2nd metatarsus			
25 Hz	0.78 ± 0.04	0.77 ± 0.02	0.75 ± 0.03
150 Hz	1.08 ± 0.04^(3)^	1.02 ± 0.08^(3)^	0.96 ± 0.06^(2)^
Heel			
25 Hz	0.84 ± 0.04	0.88 ± 0.06	0.97 ± 0.07
150 Hz	0.96 ± 0.05^(1)^	1.01 ± 0.08^(1)^	0.97 ± 0.06^(1)^
Walking			
2nd metatarsus			
25 Hz	0.78 ± 0.05	0.88 ± 0.08	0.91 ± 0.12
150 Hz	0.89 ± 0.05^(1)^	1.01 ± 0.04^(1)^	0.94 ± 0.08
Heel			
25 Hz	0.91 ± 0.04	0.88 ± 0.11	0.93 ± 0.10
150 Hz	1.09 ± 0.06^(1)^	1.09 ± 0.07^(2)^	0.96 ± 0.06

^(1)^*P* < 0.05; ^(2)^*P* < 0.01; ^(3)^*P* < 0.001

### Changes in postural control after weight carrying

After weight carrying in the static condition, the mean plantar contact area and both the peak and mean plantar pressures significantly increased, and both the COP surface and lateral deviation also significantly increased (Table 3), indicating an altered postural control.

**Table 3:** Pedobarographic measurements before (control) and after a standing upright period while carrying the added loads (mean ± SEM)

Categories	Control	Added overload
Total plantar contact area(cm^2^)	144 ± 5^(2)^	160 ± 0.9
Peak plantar pressure(N/m^2^)	16.2 ± 0.9^(2)^	18.7 ± 0.9
Mean plantar pressure(N/m^2^)	4.8 ± 0.2^(2)^	5.7 ± 0.2
COP surface(mm^2^)	137 ± 21^(1)^	187 ± 26
COP medial deviation(mm)	3.2 ± 0.3	3.7 ± 0.2
COP lateral deviation(mm)	2.1 ± 0.2^(1)^	2.8 ± 0.2

COP. Center of pressure; (1) *P* < 0.01; (2) *P* < 0.001

## Discussion

The present findings contribute to various studies that have examined the foot sole sensitivity in different contexts [6, 15–17]. Our data show that the vibration threshold was elevated only for the sole 25 Hz vibration frequency. We also reported a reduction in perceptual representation of vibration explored by the Stevens power function. Because the responses to both the lowest (25 Hz) and highest (150 Hz) vibration frequencies, and the foot sole locations, were differentially affected after weight carrying sessions when standing or walking, one may suppose that different mechanoreceptor endings are concerned. Thus, after standing with weight, the perception was altered for the sole 25 Hz frequency in both the 5th metatarsal head and the heel, suggesting a reduced activation of the Meissner corpuscles in the two foot locations. On the other hand, after walking with weight, the sole alteration of perception occurred in the 5th metatarsal head and at the 2 tested vibration frequencies. It seems that both the Meissner and Pacinian endings of the forefoot are the target of weight carrying during walking. During locomotion, the forefoot is involved in the push off phase. This is in agreement to the fact that the present results showed that a difference was observed under the heel after walking and that this increase likely targets the Meissner endings.

We also observed that after weight carrying when standing the postural sway was altered as already reported by several authors [1, 3–5]. Majumdar et al. [1] have shown that load carrying in soldiers affected kinematics of the gait. Carrying a heavy military backpack increased the postural sway during standing [3] and carrying an equipment of firefighter accentuated the gait instability [4, 5]. Park et al. [4] reported a decrease in anterior-posterior and medial-lateral excursion of the center of plantar pressure (COP) trajectory during walking with decreased COP velocity and increased foot-ground contact time and stride time. However, these data were not discussed in terms of the changes in foot sole sensitivity.

The limitation of the research is that pedobarographic measurements were not performed on the same day as the vibration perception trials because the pedobarographic platform was not available at this time. On the other hand, our study was limited to after load evaluations because the measurements of vibration sensitivity cannot be performed during weight carrying sessions. Despite the changes being significant, they cannot reflect the true effects when people carry heavy loads. We suggest that the altered vibration perception should be higher during weight carrying. However, future work is needed to evaluate the characteristics of foot sole cutaneous afferents in a loaded and/or standing posture.

The mechanism of reduced foot sole sensitivity with standing upright or walking with added weights can be only suspected. As cited above, Mildren and coworkers [16] reported elevated vibration perceptual thresholds of the heels and metatarsals in a condition of increased pressure exerted on the foot sole when sitting. We also showed that any increase in skin hardness with calluses, a condition which increased the counterpressure exerted on the foot sole, reduced the foot sole sensitivity [15]. It may be thought that any transient (weight carrying) pressure increase on the foot sole could alter its mechanosensitivity.

We partly confirmed the observations by Heller et al. [3] who showed that carrying a military backpack increased the postural sway during standing. The postural control depends on the integration of several sensory pathways, including the visual and vestibular information, the proprioceptive afferents from the neck, shoulders and limb muscles, as well as the mechanosensitivity of the foot sole [7–9]. Thus, an altered postural control after weight carrying could result from several mechanisms. The sensory pathways from the foot sole mechanoreceptors could play a key role. Indeed, it has been reported that reducing the plantar cutaneous sensation alter the walking pattern and modifies pressure distribution [22–24]. These data suggest that the overloading-induced reduction of mechanosensitive sensory pathways from the foot sole could alter the control of posture.

## Conclusion

In conclusion, heavy backpacks carried by soldiers and firefighters could impair the completion of their tasks through the reduced information carried by the afferent sensitivity from the foot sole. This is accompanied by alterations of the postural control.

## References

[1] Majumdar D, Pal MS, Majumdar D (2010). Effects of military load carrying on kinematics of gait. Ergonomics.

[2] Knapik JJ, Reynolds KL, Harman E (2004). Soldier load carrying: historical, physiological, biomechanical, and medical aspects. Mil Med.

[3] Heller ME, Challis JH, Sharkey NA (2009). Changes in postural sway as a consequence of wearing a military backpack. Gait Posture.

[4] Park H, Kim S, Morris K, Moukperian M, Moon Y, Stull J (2015). Effects of firefighters’ personal protective equipment on gait. Appl Ergon.

[5] Sako H, Kawahara M, Tanaka H (2004). The effects of the load mass and load position on body sway in supporting a load on the back. Japan Hum Ergol (Tokyo).

[6] Kim SH, Neuschwander TB, Macias BR, Bachman L, Hargens AR (2014). Upper extremity hemodynamics and sensation with backpack loads. Appl Ergon.

[7] Maurer C, Mergner T, Bolha B, Hlavacka F (2001). Human balance control during cutaneous stimulation of the plantar soles. Neurosci Lett.

[8] Meyer PF, Oddsson LI, De Luca CJ (2004). The role of plantar cutaneous sensation in unperturbed stance. Exp Brain Res.

[9] Wu G, Chiang JH (1997). The significance of somatosensory stimulations to the human foot in the control of postural reflexes. Exp Brain Res.

[10] Johansson RS, Landstrom U, Lundstrom R (1982). Responses of mechanoreceptive afferent units in the glabrous skin of the human hand to sinusoidal skin displacements. Brain Res.

[11] Strzalkowski NDJ, Ali RA, Bent LR (2017). The firing characteristics of foot sole cutaneous mechanoreceptor afferents in response to vibration stimuli. J Neurophysiol.

[12] Johansson RS, Flanagan JR (2009). Coding and use of tactile signals from the fingertips in object manipulation tasks. Nat Rev Neurosci.

[13] Kennedy PM, Inglis JT (2002). Distribution and behaviour of glabrous cutaneous receptors in the human foot sole. J Physiol.

[14] Abraira VE, Ginty DD (2013). The sensory neurons of touch. Neuron.

[15] Jammes Y, Viala M, Dutto W, Weber JP, Guieu R. Skin hardness and epidermal thickness affect the vibration sensitivity of the foot sole. Clin Res Foot Ankle. 2017;5:245. 10.4172/2329-910X.1000245.

[16] Mildren RL, Strzalkowski ND, Bent LR (2016). Foot sole skin vibration perceptual thresholds are elevated in a standing posture compare to sitting. Gait Posture..

[17] Lhomond O, Teasdale N, Simoneau M, Mouchnino L (2016). Neural consequences of increasing body weight: evidence from somatosensory evoked potentials and the frequency-specificity of brain oscillations. Front Hum Neurosci.

[18] Balzamo E, Burnet H, Zattara-Hartmann MC, Jammes Y (1995). Increasing background inspiratory resistance changes somatosensory sensations in healthy man. Neurosci Lett.

[19] Jammes Y, Guimbaud J, Faure R, Griffon P, Weber JP, Vie B (2016). Psychophysical estimate of plantar vibration sensitivity brings additional information to the detection threshold in young and elderly subjects. Clin Neurophysiol Practice.

[20] Vie B, Nester CJ, Porte LM, Behr M, Weber JP, Jammes Y (2015). Pilot study demonstrating that sole mechanosensitivity can be affected by insole use. Gait Posture..

[21] Stevens SS (1957). On the psychological law. Psychol Rev.

[22] Eils E, Behrens S, Mers O, Thorwesten L, Völker K, Rosenbaum D (2004). Reduced plantar sensation causes a caution walking pattern. Gait Posture..

[23] Eils E, Nolte S, Tewes M, Thorwesten L, Völker K, Rosenbaum D (2002). Modified pressure distribution patterns in walking following reduction of plantar sensation. J Biomech.

[24] Höhne A, Ali S, Stark C, Brüggemann GP (2012). Reduced plantar cutaneous sensation modifies gait dynamics, lower-limb kinematics and muscle activity during walking. Eur J Appl Physiol.

